# Supplementary material for: Measuring organisational readiness for health system change in South Africa using the European Foundation for Quality Management Excellence Model (EFQM)
Source: PLOS Glob Public Health. 2026 Jun 2;6(6):e0006141. doi: 10.1371/journal.pgph.0006141 (PMC13229331; doi:10.1371/journal.pgph.0006141)
Supplement: Attachment — Submitted filename: Responses to the Reviewers Final.docx [file pgph.0006141.s002.docx]

**RESPONSES TO REVIEWERS FEEDBACK AND OBSERVATIONS**

| **Reviewer Name OR Number** | **Review Feedback Number** | **Reviewer’s Query** | **Author’s Response to the Query** | **Page where Query is Addressed** | **Sentence Number Where Query is Addressed** |
| --- | --- | --- | --- | --- | --- |
| Academic Editor |  | All PLOS journals now require all data underlying the findings described in their manuscript to be freely available to other researchers, either   1. In a public repository, 2. Within the manuscript itself, or 3. Uploaded as supplementary information. | The data used for the Analysis is made available to all the reviewers in the manuscripts itself – from sentence 237 through to sentence 297. This is in line with the following statement from PLOS One – “All PLOS journals now require all data underlying the findings described in their manuscript to be freely available to other researchers, either - 2. Within the manuscript itself, | 10 – 14 | Sentence 237 through to Sentence 297. |
| Academic Editor |  | review your reference list to ensure that it is complete and correct. | The reference list is complete and correct.  Three more references were added informed by the suggestion from Reviewer #3. | 28 - 35 | Sentence 649 - 891 |
| Reviewer #1 | 1.1 | data used for the analysis should be fully made available (not at a point of request as stated in the paper). | The data used for the Analysis is made available to all the reviewers in the manuscripts itself – from sentence 237 through to sentence 297. This is in line with the following statement from PLOS One – “All PLOS journals now require all data underlying the findings described in their manuscript to be freely available to other researchers, either - 2. Within the manuscript itself, | 10 – 14 | Sentence 237 through to Sentence 297. |
|  | 1.2 | The figure titled "EFQM Domain Performance Radar Plot" after line 318 should be numbered e.g., Figure 1. | The Figure has been titled as guided. | 16 | Sentence 232 |
|  | 1.2 | Table 3 has been numbered repeatedly in line 292 and line 364. | The mistake has been picked up. This error has since been corrected.  The noted “second” Table 3 and its reference in the narrative is actually Table 4.  The narrative where the erroneous Table 3 was mentioned has since been corrected. | 14  17  18 | Sentence 294  Sentence 352  Sentence 370 |
|  | | | | | |
| Reviewer #2 | 2.1 | They are a few things that need some work: The abstract states that Leadership scored 76.9%, Policy and Strategy scored 72.8%, People scored 51.8%, and Partnerships and Resources scored 45.5%. | The Percentages have been corrected |  |  |
|  | 2.2 | Table 2 aligns with the abstract, listing Leadership at 76.94%, Policy and Strategy at 72.79%, People at 51.71%, Partnerships and Resources at 45.50%, and Processes at 63.29%. | The Percentages have been corrected to read as follows : -  Leadership scored - 76.9%,  Policy and Strategy scored - 72.8%, People scored - 51.8%,  Partnerships and Resources scored - 45.5%.  Processes scored - 63.29% |  |  |
|  | 2.3 | However, the narrative under "EFQM Component Scores" completely contradicts these figures, claiming Leadership scored 79.9%, Processes scored 79.7%, People scored 74.1%, Strategy scored 62.4%, and Partnerships & Resources scored 60.3%. | The Percentages have been corrected to read as follows : -  Leadership scored - 76.9%,  Policy and Strategy scored - 72.8%, People scored - 51.8%,  Partnerships and Resources scored - 45.5%.  Processes scored - 63.29% | 15 | Sentences 297 through 313, and |
|  | 2.5 | The "EFQM Domain Performance Radar Plot" visually and textually supports the incorrect narrative numbers rather than the primary data in Table 2. | The "EFQM Domain Performance Radar Plot"  Leadership scored - 76.9%,  Policy and Strategy scored - 72.8%, People scored - 51.8%,  Partnerships and Resources scored - 45.5%.  Processes scored - 63.29% | 16 | Sentence 321 |
|  | 2.6 | There is a sequencing error with the tables; the designation "Table 3" is used twice for entirely different datasets (once for organizational maturity comparisons and once for multivariate associations). | The mistake has been picked up. This error has since been corrected.  The noted “second” Table 3 and its reference in the narrative is actually Table 4.  The narrative where the erroneous Table 3 was mentioned has since been corrected. | 14  17  18 | Sentence 294  Sentence 352  Sentence 370 |
|  | 2.7 | An article by Oleribe et al. has the wrong reference year (2029, instead of 2019). | Referencing has been corrected. Year of the citation has been corrected to 2019. | 28 | Sentence 669 |
|  | | | | | |
| Reviewer #3 | 3.1 | As a minor suggestion, the authors could briefly expand on how EFQM domains might be adapted or operationalised differently within resource-constrained public health systems, as this would further enhance the paper’s relevance to other low- and middle-income contexts. | The “minor” suggestion has been accepted and acted upon. A new paragraph has been added to the document. | 24 | Sentences  542-566 |
|  | | | | | |
