# Supplementary material for: Measuring organisational readiness for health system change in South Africa using the European Foundation for Quality Management Excellence Model (EFQM)
Source: PLOS Glob Public Health. 2026 Jun 2;6(6):e0006141. doi: 10.1371/journal.pgph.0006141 (PMC13229331; doi:10.1371/journal.pgph.0006141)
Supplement: Attachment — Submitted filename: 17_04_2026 Response to the Reviewer v3.docx [file pgph.0006141.s003.docx]

RESPONSES TO THE REVIEWERS

| **Reviewer Number** | **Query number** | **Query** | **Response in full** | **Line and page number** |
| --- | --- | --- | --- | --- |
| 3 | 1 | Data Mismatch (Partnerships and Resources) discussion – re: 63.29% vs 45.50% | The error is conformed and regretted. This error has since been resolved.  45.50% has been inserted to replace 63.29% | Page Number 22, Line Number 501 |
| 3 | 2 | Mischaracterisation of Domain Ranking (EFQM Compound Scores section) – The text states that “Strategy (72,79%) and Partnerships & Resources (45.50%) were the two lowest scoring domains | The error is conformed and regretted. This error has since been resolved.  People (51.81%) has been inserted replacing Strategy (72.79%) | Page Number 15, Line Number 307 |
| 3 | 3a | Reference alignment in the new limitation paragraph. The citation to reference (26) on mitigating EFQM implementation costs may not match the referenced source(Toth and Szucs, 2025 about EFQM and leadership) | The error is confirmed. Reference 26 has been corrected. | Page Number 30, Line Number 767 – 769. |
|  | 3b | Reference alignment in the new limitation paragraph. The citation to reference (26) on mitigating EFQM implementation costs may not match the referenced source(Toth and Szucs, 2025 about EFQM and leadership) | The subject of “mitigating EFQM implementation costs – the attribution is indeed incorrect. This has now been corrected- the citation is from an article by Shafiq (2012) and . The sentence is also rephrased for a better citation | Correction 1  Page Number 24 and Line Number 571  Correction 2  Page Number 34, Line Number 884 - 886 |
| 3 | 4 | Minor editorial items   i. “apar” sometimes “PR” – ii. Decimal Notations are inconsistent | i. Error is regretted and has been corrected. ii. Error is confirmed and regretted. Decimal Notations have been corrected | Page Number 17, Line Numbers – 355 to 367 |
| 4 | 1 | All data used to arrive at the findings and conclusions will be made available upon request | This is an error – this will be corrected at resubmission | Not Applicable |
|  | 2 | **Clarity regarding EFQM** |  | Page Number 2  Line Number 25  Page Numbers – 4 and 5  Line Numbers 104 to 130 |
|  | 3 | Spell Out EFQM in full at first mention | EFQM as an Abbreviation and Written out in full in the Abstract section to address this observation and guidance - |  |
|  | 4 | Provide additional context on its purpose and core domains | Additional Context has been provided |  |
|  | 5 | Why EFQM is an appropriate framework for assessing health systems | This enquiry is addressed. |  |
|  | 6 | Any adaptations made to apply the framework in this context should clearly be described |  |  |
|  | 7 | **Table 2 would benefit from improved formatting** |  | Page Number 11 and 12  Line Numbers – 285 to 286 |
|  |  | Current layout | The Table has been changed to take away all the colours. It is now plain white. The content is now aligned to the Left |  |
|  |  | Spacing | The line spacing is 1.0 across the whole Table |  |
|  |  | Section breaks | The removal of the colours makes the section breaks easier to discern |  |
|  | 8 | The Authors state that “all data used to arrive at the findings and conclusions will be made available” – This approach does not align with PLOS guidelines, which require that data be made openly available without restriction. The Authors should ensure compliance with the journal’s data sharing policy as applicable. | The insertion in this area has been amended to read as follows – “All relevant data underlying the findings is included in the articles and is therefore made fully available and without any restriction. We conform that we are fully committed to comply with the journal’s data sharing policy as applicable. | Not Applicable |
